# The UCSC Genome Browser database: 2023 update

**DOI:** 10.1093/nar/gkac1072

**Published:** 2022-11-24

**Authors:** Luis R Nassar, Galt P Barber, Anna Benet-Pagès, Jonathan Casper, Hiram Clawson, Mark Diekhans, Clay Fischer, Jairo Navarro Gonzalez, Angie S Hinrichs, Brian T Lee, Christopher M Lee, Pranav Muthuraman, Beagan Nguy, Tiana Pereira, Parisa Nejad, Gerardo Perez, Brian J Raney, Daniel Schmelter, Matthew L Speir, Brittney D Wick, Ann S Zweig, David Haussler, Robert M Kuhn, Maximilian Haeussler, W James Kent

**Affiliations:** Genomics Institute, University of California Santa Cruz, Santa Cruz, CA 95064, USA; Genomics Institute, University of California Santa Cruz, Santa Cruz, CA 95064, USA; Institute of Neurogenomics, Helmholtz Zentrum München GmbH - German Research Center for Environmental Health, 85764Neuherberg, Germany; Medical Genetics Center (Medizinisch Genetisches Zentrum), Munich 80335, Germany; Genomics Institute, University of California Santa Cruz, Santa Cruz, CA 95064, USA; Genomics Institute, University of California Santa Cruz, Santa Cruz, CA 95064, USA; Genomics Institute, University of California Santa Cruz, Santa Cruz, CA 95064, USA; Genomics Institute, University of California Santa Cruz, Santa Cruz, CA 95064, USA; Genomics Institute, University of California Santa Cruz, Santa Cruz, CA 95064, USA; Genomics Institute, University of California Santa Cruz, Santa Cruz, CA 95064, USA; Genomics Institute, University of California Santa Cruz, Santa Cruz, CA 95064, USA; Genomics Institute, University of California Santa Cruz, Santa Cruz, CA 95064, USA; Genomics Institute, University of California Santa Cruz, Santa Cruz, CA 95064, USA; Genomics Institute, University of California Santa Cruz, Santa Cruz, CA 95064, USA; Genomics Institute, University of California Santa Cruz, Santa Cruz, CA 95064, USA; Genomics Institute, University of California Santa Cruz, Santa Cruz, CA 95064, USA; Genomics Institute, University of California Santa Cruz, Santa Cruz, CA 95064, USA; Genomics Institute, University of California Santa Cruz, Santa Cruz, CA 95064, USA; Genomics Institute, University of California Santa Cruz, Santa Cruz, CA 95064, USA; Genomics Institute, University of California Santa Cruz, Santa Cruz, CA 95064, USA; Genomics Institute, University of California Santa Cruz, Santa Cruz, CA 95064, USA; Genomics Institute, University of California Santa Cruz, Santa Cruz, CA 95064, USA; Genomics Institute, University of California Santa Cruz, Santa Cruz, CA 95064, USA; Genomics Institute, University of California Santa Cruz, Santa Cruz, CA 95064, USA; Genomics Institute, University of California Santa Cruz, Santa Cruz, CA 95064, USA; Genomics Institute, University of California Santa Cruz, Santa Cruz, CA 95064, USA

## Abstract

The UCSC Genome Browser (https://genome.ucsc.edu) is an omics data consolidator, graphical viewer, and general bioinformatics resource that continues to serve the community as it enters its 23rd year. This year has seen an emphasis in clinical data, with new tracks and an expanded Recommended Track Sets feature on hg38 as well as the addition of a single cell track group. SARS-CoV-2 continues to remain a focus, with regular annotation updates to the browser and continued curation of our phylogenetic sequence placing tool, hgPhyloPlace, whose tree has now reached over 12M sequences. Our GenArk resource has also grown, offering over 2500 hubs and a system for users to request any absent assemblies. We have expanded our bigBarChart display type and created new ways to visualize data via bigRmsk and dynseq display. Displaying custom annotations is now easier due to our chromAlias system which eliminates the requirement for renaming sequence names to the UCSC standard. Users involved in data generation may also be interested in our new tools and trackDb settings which facilitate the creation and display of their custom annotations.

## INTRODUCTION

The University of California Santa Cruz (UCSC) Genome Browser (1) is an online resource for the genomics community providing data access and visualization, collaboration and support resources, and a suite of tools that are now standard in the field. With the ever-increasing amounts of data being generated every year, tools like the UCSC Genome Browser and other browsers (2–6) are increasingly playing a key step in analysis and interpretation. Our resource services over 1.4 million users per year across its primary site as well as its European and Asian based mirrors. We also maintain near 100% uptime and continually update our software on a tri-week cycle.

With regards to data access and visualization, we offer over 6000 tracks on the two latest human GRCh assemblies alone, GRCh38/hg38 and GRCh37/hg19. There are also over 200 assemblies available on the Genome Browser and over 2000 if GenArk (7) is included. We support over 30 data formats such as bed/bigBed, wig/bigWig (8), VCF (9) and GTF/GFF. This not only allows users to display their own annotations, but also to visualize data from a large number of sources in a single location. Nearly all data is available for extraction via bulk download, public MySQL server, RESTful API (10) or the Table Browser (11).

We also provide tools to facilitate scientific collaboration as well as support for the community. Immutable snapshots of annotations and locations can be shared via the sessions feature (My Data → My Sessions), custom data can be shared as custom tracks (My Data → Custom Tracks) and hubs (My Data → Track hubs), and user-generated hubs can be shared with the wider community by means of the Public Hub list. We also respond to over 600 mailing list questions per year, assisting users with topics such as how best to display their data, troubleshooting our tools, and generating chain files for lifting between assemblies.

Lastly, we provide and support many other tools and utilities. Some of the most popular tools not yet mentioned are BLAT (12) for placing sequences, In-Silico PCR for identifying PCR primers, and LiftOver which provides a web interface for converting genomic coordinates between assemblies. Our hundreds of utilities (https://hgdownload.soe.ucsc.edu/downloads.html#utilities_downloads) can also be downloaded. These include file format creation, such as bedToBigBed, command line versions of our web tools such as liftOver, and other resources. And for users that may have sensitive data or poor connections, we offer various ways to mirror our software locally (Mirrors → Mirroring Instructions). For more information on what the Genome Browser has to offer, visit our training page (https://genome.ucsc.edu/training).

## NEW AND UPDATED ANNOTATIONS

Over the last year we have added and updated over 50 annotation tracks to existing assemblies, added a new single-cell annotation group to hg38, made seven new or updated Public Hubs available, and added over 900 new assembly hubs via GenArk. We have also created hundreds of liftOver files, all of which are available on our download server, which allow coordinate lifting between assemblies. This includes 36 files directly requested by users on our mailing list.

### New clinical data

Twelve new tracks have been added to human assemblies in support of variant interpretation and clinical genomics. Some notable examples include DECIPHER (DatabasE of genomiC varIation and Phenotype in Humans using Ensembl Resources) (13), which aggregates variant information from various sources, added to hg38; Orphanet (14), which provides comprehensive datasets related to rare diseases and orphan drugs from the Orphanet knowledge base; GenCC (The Gene Curation Coalition) (15), which aims to collect and standardize gene-disease validity annotations across various submitters; and dbSNP155 (16), which is the latest NCBI dbSNP release with over one billion variants. We have also continued to update our Microarray Probesets tracks, which now contain the positions of probes and targets of over 50 NGS arrays. A new Constraint Scores track is also available which hosts various mutation constraint annotations from different data providers. For a complete list of new and updated clinical tracks see Supplementary Table S1.

**Figure 1.**
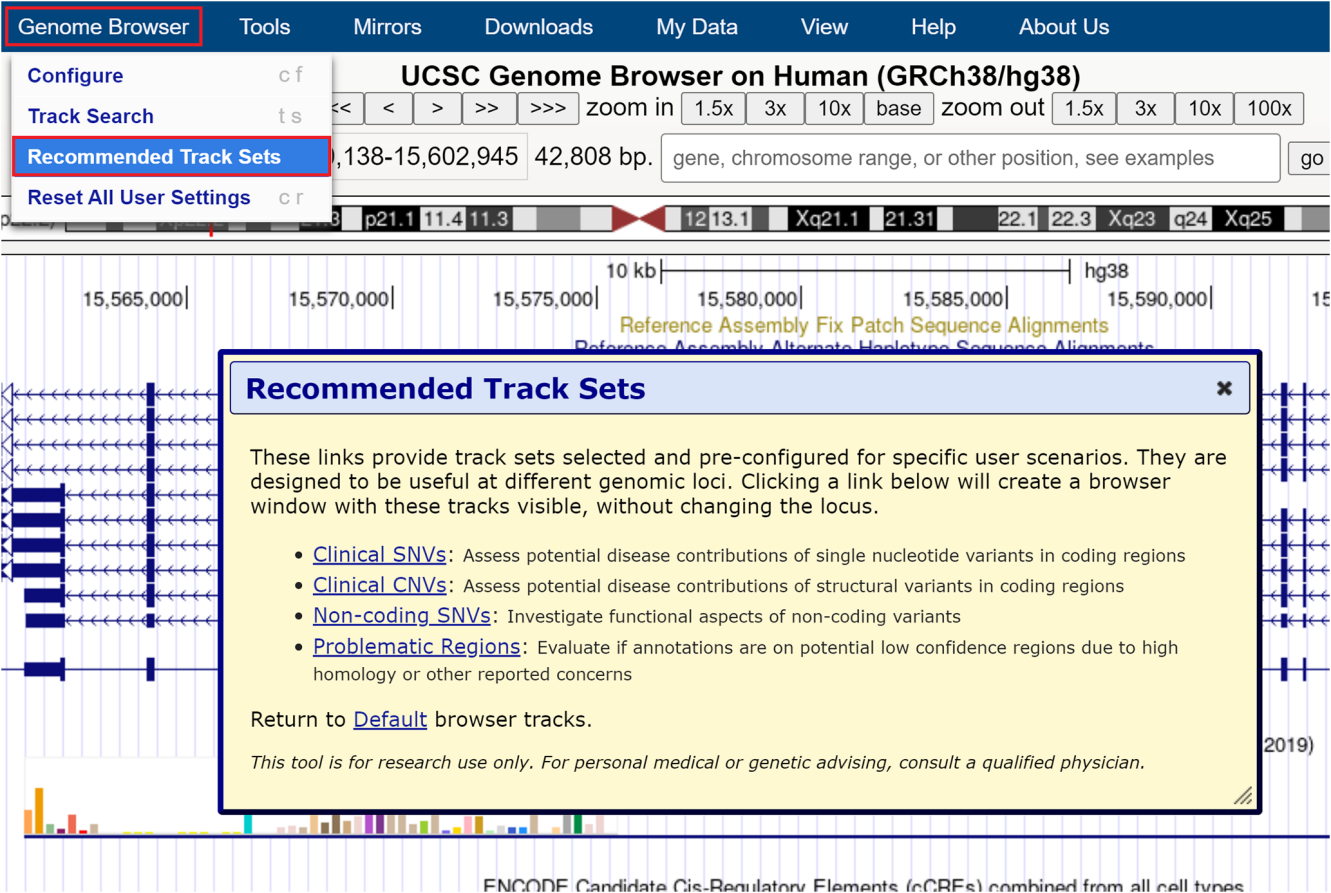
Recommended Track Sets available for hg38 in the Genome Browser menu (Genome Browser → Recommended Track Sets).

In order to better introduce the clinical resources available on hg38, we have expanded our Recommended Track Sets (17) (https://genome.ucsc.edu/goldenPath/newsarch.html#022222) feature to hg38 (Figure 1). Like hg19, this feature contains 4 sets of curated track configurations for different clinical applications.

### New single-cell track group on hg38

**Figure 2.**
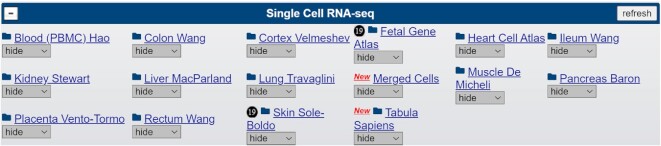
Single-cell RNA-seq track group now available on hg38.

We have added a new single-cell RNA-seq (scRNA-seq) track group to hg38 (Figure 2). It currently contains 14 scRNA-seq tracks, originally wrangled into our Cell Browser (https://cells.ucsc.edu) (18), covering major organs of the body with each track being comprised of 2–19 individual mRNA expression tracks in barChart format. There are also two aggregate tracks: Tabula Sapiens (19), which contains data from the Tabula Sapiens Consortium providing an atlas of nearly 500 000 cells from 24 organs of 15 normal humans, and a Merged Cells track, which is an aggregate track created by the Genome Browser containing data from 12 papers covering 14 organs. A complete list of new single-cell tracks is available in Supplementary Table S2.

### Gene set updates

This year we have added or updated 15 gene annotations for human and mouse. We continue to provide the latest GENCODE gene models (20), currently v41, which are always available on hg38, hg19 and mm39. We also archive these releases for reproducibility, having added 38–41 during this period. The NCBI RefSeq gene models (21) on hg38 and hg19 have also been updated corresponding to NCBI release 109.20211119 and 105.20220307 respectively. We have also added the 1.0 release of the Matched Annotation from NCBI and EMBL-EBI (MANE) project (22), which provides a set of high-confidence transcripts that are identically annotated between RefSeq and Ensembl/GENCODE. Lastly, we have updated select tables (kgXref, kgAlias) and our search files for the default hg19 gene annotation track UCSC Genes (knownGene) (23) so that new and updated gene symbols can be found.

### Other new tracks

In addition to clinical, single-cell and gene tracks, we have added 8 new tracks to our vertebrate assemblies. These include a European Variation Archive (EVA) (24) track corresponding to EVA release 3 on 14 assemblies including mm39, providing novel variant data on these Browsers. There is also now a 241-way Cactus (25,26) comparative genomics alignment track on hg38 generated by the Zoonomia Project (27), which is the largest conservation track in the Genome Browser. We have also added various regulatory tracks and additional annotations from the GTEx Consortium (28). See Supplementary Tables S2–S4 for a full list of tracks and assemblies. We also continue to run our pipelines which automatically update annotations on 13 tracks, which can be seen in Supplementary Table S5.

### SARS-CoV-2 genome browser updates

We continue to regularly update our SARS-CoV-2 assembly data, adding or updating 14 tracks over the last year. Among these tracks is our Variants of Concern (VOC) track, which we continue to update with the latest WHO-designated variants of concern. For a full list of updated tracks see Supplementary Table S4.

Curation has also been ongoing on the growing phylogenetic tree which supports our tool for placing SARS-CoV-2 sequences using UShER (29), hgPhyloPlace (https://genome.ucsc.edu/cgi-bin/hgPhyloPlace). The tree now contains over 12 million sequences, with updates occurring daily. A minimized version of the tree is included in the pangolin tool (30), used by public health departments worldwide to assign lineages to new sequences. The full tree including GISAID sequences cannot be redistributed due to GISAID restrictions (www.gisaid.org), but we offer download files for a public sequence tree with over 6 million sequences (https://hgdownload.gi.ucsc.edu/goldenPath/wuhCor1/UShER_SARS-CoV-2/) (31). We have also recently expanded hgPhyloPlace for use with monkeypox (RefSeq NC_063383.1).

### New hubs

This year we have added 7 new ‘Public hubs’, which are externally hosted and maintained annotations available to our users via the Track Data Hubs page (https://genome.ucsc.edu/cgi-bin/hgHubConnect). We continue to accept submissions from users looking to promote and share their data. These new hubs include the 2022 update of the popular ReMap Regulatory Atlas hub (32), which contains transcriptional regulator annotations on 6 model genomes, and a 605 species Mammal and Bird alignment using the Cactus aligner. For a full list see Supplemental Table S6.

## NEW ASSEMBLY DATA

Over the last year we have updated the official patch sequences from the Genome Reference Consortium (GRC) for hg38 and mm10. The GRCh38/hg38 assembly has been updated to patch 13, and GRCm38/mm10 has been updated to patch 6. These updates contain both fix sequences and alternate haplotypes.

### Genome Archive (GenArk)

With the continued drop in sequencing cost and increase in assembly quality, we have expanded the resources spent on rapid creation of browsers via assembly hubs based on GenBank (33) assembly accessions. This collection of in-house generated hubs, referred to as Genome Archive (GenArk - https://hgdownload.soe.ucsc.edu/hubs/), currently contains 2589 hubs. Over the last year alone we have added 904 new NCBI/VGP assemblies. There is now also a viral genomes category (https://hgdownload.soe.ucsc.edu/hubs/viral/index.html) containing 257 viral assemblies ready for display. In response to user demand, we have created an assembly request page (https://genome.ucsc.edu/assemblyRequest.html). This page allows users to search for most GenBank assemblies, currently containing 15 018 eligible browser candidates, and request a browser be created if one does not already exist. New browsers are typically ready in less than a week. For more information on GenArk, see our detailed four-part blog series on the topic (https://genome-blog.soe.ucsc.edu/blog/2021/11/23/genark-hubs-part-1/).

### T2T CHM13 v2.0 assembly (hs1)

Soon after the T2T consortium published their T2T CHM13 v2.0 assembly (34), we created a GenArk browser to display the sequence alongside various annotation tracks which were a combination of consortium-generated and in-house data. These include various gene annotations, lifted clinical data, and comparative genomics tracks focused on the new sequence added in T2T CHM13 v2.0. We expanded our hgConvert (https://genome.ucsc.edu/cgi-bin/hgConvert?db=hg38) and hgLiftOver (https://genome.ucsc.edu/cgi-bin/hgLiftOver?db=hg38) tools to support GenArk assemblies in order to facilitate data conversion between hg19/hg38 and T2T CHM13 v2.0.

In anticipation of many high-quality genomes becoming available in the near future, T2T CHM13 v2.0 was the first human assembly to be elevated from hub to curated hub. Curated hubs, while still hubs, have all the support of native assemblies such as easier discovery and track search, API support, and the ability to add custom annotations without first having to connect to the hub. With this change to curated hub the assembly name was changed to *Homo sapiens* 1 (hs1). T2T CHM13 v2.0 (hs1) can be accessed directly from the Genomes dropdown menu. It is worth noting that to users curated hubs are functionally identical to native assemblies (e.g. hg19, hg38), and that in line with our reproducibility practice, all previous hubs and hub data will continue to exist.

## NEW GENOME BROWSER SOFTWARE

**Figure 3.**
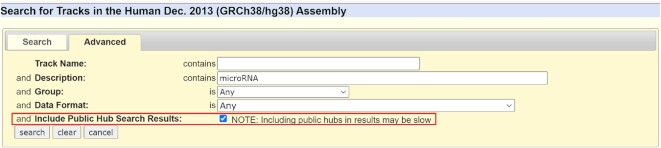
Advanced options in Track Search now includes the option to search Public Hub tracks.

Over the last year we have expanded functionality of the Genome Browser with small additions as well as new and updated displays and settings. By user request, we have added a comma separated values option to the Table Browser output. The new setting can be toggled on the ‘output field separator’ tab and facilitates data download for use in other software such as excel. It is now also possible to include Public Hub tracks in the Track Search (https://genome.ucsc.edu/cgi-bin/hgTracks?db=hg38&hgt_tSearch=track+search) results by toggling on the feature in the advanced options (Figure 3).

### New displays

#### bigBarChart

**Figure 4.**
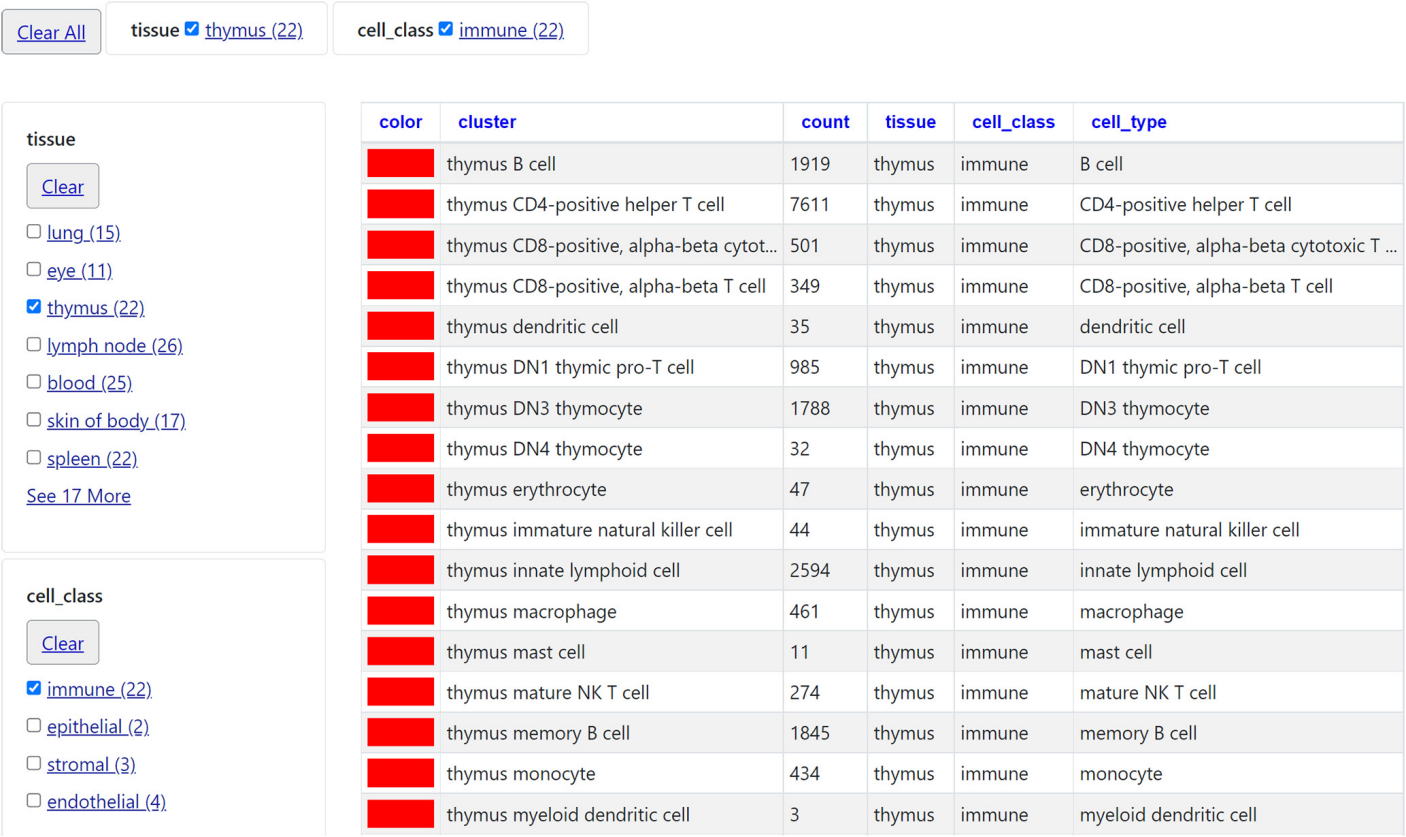
Search facets for bigBarChart track Tabula Sapiens: Tabula Tissue Cell.

Two new settings have been added to the bigBarChart (https://genome.ucsc.edu/goldenPath/help/barChart.html) format to allow for additional customization of how the bars display: barChartBarMinWidth and barChartBarMinPadding. There is also a new feature for bigBarChart tracks that enables a facet display (Figure 4) in the item details page and track configuration page (https://genome.ucsc.edu/cgi-bin/hgTrackUi?db=hg38&c=chrX&g=tabulaSapiensTissueCellType). These facets allow for visualization and grouping of complex and expansive data, such as single cell data, into various categories and granularities based on associated metadata. The facets are enabled by adding the new trackDb settings barChartFacets and barChartStatsUrl (https://genome.ucsc.edu/goldenPath/help/barChart.html#example6).

#### bigRmsk

**Figure 5.**
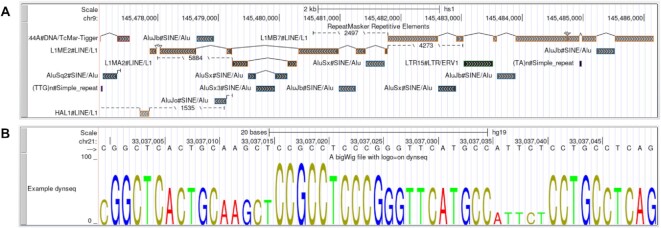
(**A**) Full display mode in bigRmsk track. (**B**) dynseq display in full mode.

The bigRmsk track type (https://genome.ucsc.edu/goldenPath/help/bigRmsk.html) has been added for displaying repeat annotations generated by the RepeatMasker program. The setting is optimized for displaying repeat types, automatically changing its display based on the window size. The track includes item coloring based on the classification of the repeat, and the Full mode includes additional details such as length of unaligned repeat model sequence and context for where a repeat fragment originates (Figure 5A).

#### dynseq display

We have added support for the dynseq display (35) developed by the Kundaje lab (https://kundajelab.github.io/dynseq-pages/). This display scales the height of each nucleotide letter based on the signal value within a bigWig track (https://genome.ucsc.edu/goldenPath/help/bigWig.html#Ex4; Figure 5B).

### New hub features and TrackDb statements

In order to facilitate custom annotations and user content, we have expanded custom track as well as hub support and added 15 new trackDb settings with various functions (Table 1). When creating hub tracks for a genome that is included in GenArk, you can now designate the GCA/GCF identifier and the Genome Browser will automatically attach the matching GenArk assembly hub genome and display the data on it (https://genome.ucsc.edu/FAQ/FAQlink.html#genArkTrackHub). This harmonizes the system to function like native assemblies, such as hg19 and hg38, and removes the requirement of a multi-line genome stanza. Another new feature that builds upon hub annotations which are designated by the bigDataUrl setting is access to the extended case/color options. This means that when browsing the tracks display while displaying hub data, you can go to View → DNA in the top blue bar menu and select the ‘extended case/color options’ button. In that page you will be able to modify the DNA sequence in the window in various ways depending on the data tracks which are currently being displayed, such as adding a specific color for any part of the sequence covered by the annotations.

**Table 1.** List of new trackDb settings added to the Hub Track Database Definition document (https://genome.ucsc.edu/goldenPath/help/trackDb/trackDbHub.html) over the last year

Setting name	Description
otherTwoBitUrl	For in pairwise alignment tracks (chain, PSL), used to specify location of query sequence.
logo	Enables the dynseq display feature on wiggle tracks.
speciesLabels	Allows one to specify new labels that map to sequence names in bigMaf tracks.
hicDistanceMax	Controls the maximum interaction distance in nucleotides for the heatmap in Hi-C tracks.
hicDistanceMin	Controls the minimum interaction distance in nucleotides for the heatmap in Hi-C tracks.
barChartFacets	Enables the facets feature in bigBarChart track description and item details pages.
barChartStatsUrl	Associates a table in tab-separated-values with the bigBarChart track, with one line per bar. Currently used in coordination with the barChartsFacets tag to specify metadata such as cell types or tissue of origin.
barChartBarMinPadding	Sets the minimum pixel width between bars for bigBarChart tracks.
barChartBarMinWidth	Sets the minimum pixel width of the bars in bigBarChart tracks.
barChartStretchToItem	Extends the barCharts to cover the entire horizontal space available in the graph. Useful for bigBarChart tracks with many bars.
pslSequence	Specifies display configuration options for PSL tracks that also have sequence loaded.
showCdsAllScales	Shows CDS for PSL tracks at all zoom levels.
showCdsMaxZoom	Specifies (bases/pixel) the maximum zoom-out allowed for displaying the CDS for PSL tracks.
showDiffBasesMaxZoom	Shows annotations highlighting base or codon differences only if current zoom level does not exceed value (bases/pixel) in PSL tracks.

#### chromAlias

The chromAlias system provides an index of corresponding sequence names across different groups and consortiums. An example would be how UCSC names chromosomes with the ‘chr’ prefix while other groups such as Ensembl (36) list only the number: ‘chr2’ in UCSC corresponds to ‘2’ in Ensembl. In the past users would have to modify the sequence names if they did not adhere to the UCSC convention, but that is no longer the case. chromAlias associations have been built for all native Genome Browsers as well as GenArk assemblies, looking for corresponding matches in GenBank, Ensembl, and RefSeq when available. When custom annotations are now attached, if the sequence names do not match UCSC’s, then the chromAlias table is referenced and displays the annotations if a match is found. This support has also been extended to the bedToBigBed utility, which now optionally accepts a chromAlias file instead of a chrom.sizes file and will build the bigBed without any need for renaming sequences. These chromAlias files can be found on our download server, e.g. hg38 (https://hgdownload.soe.ucsc.edu/goldenPath/hg38/bigZips/hg38.chromAlias.bb).

### TrackDb settings

We added 15 new trackDb settings to our Hub Track Database Definition document (https://genome.ucsc.edu/goldenPath/help/trackDb/trackDbHub.html). These include additional configurations for Hi-C track display, bigBarChart display and PSL display among others. See Table 1 for a full list and short description.

### New and updated tools

We continue to add to and maintain our suite of over 300 command line tools (https://hgdownload.soe.ucsc.edu/downloads.html#utilities_downloads). Of notable mention is our chromToUcsc tool, as well as four new tools which help with data analysis and track creation. Many were developed to assist in the creation of the new bigBarChart single cell tracks. See our bigBarChart documentation for an example (https://genome.ucsc.edu/goldenPath/help/barChart.html#example7).

#### chromToUcsc

Can be used to convert most standard annotation file formats (e.g. BED, wiggle, GTF, VCF, etc.) that contain different sequence names to those expected by UCSC, to the UCSC convention.

#### tabToTabDir

Takes a single large table and converts it to a directory full of smaller tables. This is useful for exploring and curating large metadata tables. It can be particularly helpful in reducing a table with many fields into a few normalized (in the relational sense) tables with fewer fields.

#### matrixClusterColumns

Converts a single cell gene expression matrix to a cell-type gene expression matrix. It takes a cell-by-cell metadata matrix that refers to the same cells as a gene expression matrix and combines the gene expression values for all cells of a given type into a single value representing the cell type. It can also be used on other metadata fields to produce matrices that show mean or average gene expression levels for a donor, an organ, or any other metadata field or combination of fields.

#### gencodeVersionForGenes

Takes a list of gene symbols or gene accessions and searches for the version of GENCODE or RefSeq that matches the most genes in the list. Optionally produces a bed file containing the gene structures for the genes in the list.

#### matrixToBarChartBed

Combines an expression matrix and a bed file with gene structures to make a bed file with a bar chart showing gene expression on the Genome Browser.

## OUTREACH AND CONTACT INFORMATION

The Genome Browser supports users in a variety of ways including a blog (https://genome.ucsc.edu/blog), videos (https://bit.ly/ucscVideos), and both virtual and in-person trainings (https://bit.ly/ucscTraining). In the year since the last NAR update, we have conducted 25 workshops and courses, including several at international meetings. Our training page (https://genome.ucsc.edu/training) provides access to these resources as well as an index to user guides and help pages for all the Genome Browser tools. Three new videos have also been added to the YouTube channel (https://bit.ly/ucscVideos) featuring the use of the SARS-CoV-2 browser.

We provide email support through a public and a private mailing list where users can avail themselves of our expert and responsive staff. Access to the mailing lists can be found at https://genome.ucsc.edu/contacts.html, where there is also a link to an archive of previously answered questions from the public list.

In response to inquiries from our users, we released a module of content designed for use in the undergraduate classroom. This content features vignettes written by undergraduates to illustrate, using the Genome Browser, a variety of lessons in Molecular Biology, Genetics, Medicine, Population Biology and Evolution. This can be found at https://genome.ucsc.edu/training/education.

## FUTURE PLANS

This coming year represents the first in our new 5-year planning cycle. A major goal during this time is evaluation and adoption of a pangenome graph data format. We will also be releasing a new site-wide search function and a track duplication feature. Work continues to expand hub support. Most new data will be created in big formats and new assemblies will be implemented as hubs instead of SQL databases (e.g. hs1). Along those lines, work will begin on a tool to facilitate hub development. Lastly, an emphasis on clinical genomics and single cell data will continue, with features such as Recommended Track Sets and the new single cell track group seeing updates throughout the year.

## DATA AVAILABILITY

The UCSC Genome Browser (https://genome.ucsc.edu/) is freely available to all users. The only exceptions are the source code for the Genome Browser, Blat utility, liftOver utility and other utilities which are free for non-profit academic research and for personal use. A license is required for commercial use of these utilities or the source code.

## Supplementary Material

gkac1072_Supplemental_File

## Notes

Present address: Luis Nassar, Genomics Institute, University of California Santa Cruz, Santa Cruz, CA 95064, USA.

## References

[1] Kent W.J. , Sugnet C.W., Furey T.S., Roskin K.M., Pringle T.H., Zahler A.M., Haussler D. The human genome browser at UCSC. Genome Res. 2002; 12:996–1006. 12045153 10.1101/gr.229102 PMC186604

[2] Cunningham F. , Allen J.E., Allen J., Alvarez-Jarreta J., Amode M.R., Armean I.M., Austine-Orimoloye O., Azov A.G., Barnes I., Bennett R. et al. Ensembl 2022. Nucleic Acids Res. 2022; 50:D988–D995. 34791404 10.1093/nar/gkab1049 PMC8728283

[3] Thorvaldsdóttir H. , Robinson J.T., Mesirov J.P. Integrative genomics viewer (IGV): high-performance genomics data visualization and exploration. Brief. Bioinform. 2013; 14:178–192. 22517427 10.1093/bib/bbs017 PMC3603213

[4] Li D. , Purushotham D., Harrison J.K., Hsu S., Zhuo X., Fan C., Liu S., Xu V., Chen S., Xu J. et al. WashU epigenome browser update 2022. Nucleic Acids Res. 2022; 50:W774. 35412637 10.1093/nar/gkac238 PMC9252771

[5] Buels R. , Yao E., Diesh C.M., Hayes R.D., Munoz-Torres M., Helt G., Goodstein D.M., Elsik C.G., Lewis S.E., Stein L. et al. JBrowse: a dynamic web platform for genome visualization and analysis. Genome Biol. 2016; 17:66. 27072794 10.1186/s13059-016-0924-1 PMC4830012

[6] Rangwala S.H. , Kuznetsov A., Ananiev V., Asztalos A., Borodin E., Evgeniev V., Joukov V., Lotov V., Pannu R., Rudnev D. et al. Accessing NCBI data using the NCBI sequence viewer and genome data viewer (GDV). Genome Res. 2021; 31:159–169. 33239395 10.1101/gr.266932.120 PMC7849379

[7] Lee B.T. , Barber G.P., Benet-Pagès A., Casper J., Clawson H., Diekhans M., Fischer C., Gonzalez J.N., Hinrichs A.S., Lee C.M. et al. The UCSC genome browser database: 2022 update. Nucleic Acids Res. 2021; 50:D1115–D1122. 10.1093/nar/gkab959 PMC8728131 34718705

[8] Kent W.J. , Zweig A.S., Barber G., Hinrichs A.S., Karolchik D BigWig and bigbed: enabling browsing of large distributed datasets. Bioinformatics. 2010; 26:2204–2207. 20639541 10.1093/bioinformatics/btq351 PMC2922891

[9] Danecek P. , Auton A., Abecasis G., Albers C.A., Banks E., DePristo M.A., Handsaker R.E., Lunter G., Marth G.T., Sherry S.T. et al. The variant call format and VCFtools. Bioinforma. Oxf. Engl. 2011; 27:2156–2158. 10.1093/bioinformatics/btr330 PMC3137218 21653522

[10] Lee C.M. , Barber G.P., Casper J., Clawson H., Diekhans M., Gonzalez J.N., Hinrichs A.S., Lee B.T., Nassar L.R., Powell C.C. et al. UCSC genome browser enters 20th year. Nucleic Acids Res. 2020; 48:D756–D761. 31691824 10.1093/nar/gkz1012 PMC7145642

[11] Karolchik D. , Hinrichs A.S., Furey T.S., Roskin K.M., Sugnet C.W., Haussler D., Kent W.J. The UCSC table browser data retrieval tool. Nucleic Acids Res. 2004; 32:D493–D496. 14681465 10.1093/nar/gkh103 PMC308837

[12] Kent W.J. BLAT—The BLAST-Like alignment tool. Genome Res. 2002; 12:656–664. 11932250 10.1101/gr.229202 PMC187518

[13] Firth H.V. , Richards S.M., Bevan A.P., Clayton S., Corpas M., Rajan D., Vooren S.V., Moreau Y., Pettett R.M., Carter N.P. DECIPHER: database of chromosomal imbalance and phenotype in humans using ensembl resources. Am. J. Hum. Genet. 2009; 84:524–533. 19344873 10.1016/j.ajhg.2009.03.010 PMC2667985

[14] Pavan S. , Rommel K., Mateo Marquina M.E., Höhn S., Lanneau V., Rath A. Clinical practice guidelines for rare diseases: the orphanet database. PLoS One. 2017; 12:e0170365. 28099516 10.1371/journal.pone.0170365 PMC5242437

[15] DiStefano M.T. , Goehringer S., Babb L., Alkuraya F.S., Amberger J., Amin M., Austin-Tse C., Balzotti M., Berg J.S., Birney E. et al. The gene curation coalition: a global effort to harmonize gene–disease evidence resources. Genet. Med. 2022; 24:1732–1742. 35507016 10.1016/j.gim.2022.04.017 PMC7613247

[16] Sherry S.T. , Ward M.-H., Kholodov M., Baker J., Phan L., Smigielski E.M., Sirotkin K. dbSNP: the NCBI database of genetic variation. Nucleic Acids Res. 2001; 29:308–311. 11125122 10.1093/nar/29.1.308 PMC29783

[17] Benet-Pagès A. , Rosenbloom K.R., Nassar L.R., Lee C.M., Raney B.J., Clawson H., Schmelter D., Casper J., Gonzalez J.N., Perez G. et al. Variant interpretation: UCSC genome browser recommended track sets. Hum. Mutat. 2022; 43:998–1011. 35088925 10.1002/humu.24335 PMC9288501

[18] Speir M.L. , Bhaduri A., Markov N.S., Moreno P., Nowakowski T.J., Papatheodorou I., Pollen A.A., Raney B.J., Seninge L., Kent W.J. et al. UCSC cell browser: visualize your single-cell data. Bioinformatics. 2021; 37:4578–4580. 34244710 10.1093/bioinformatics/btab503 PMC8652023

[19] Schaum N. , Karkanias J., Neff N.F., May A.P., Quake S.R., Wyss-Coray T., Darmanis S., Batson J., Botvinnik O., Chen M.B. et al. Single-cell transcriptomics of 20 mouse organs creates a tabula muris. Nature. 2018; 562:367–372. 30283141 10.1038/s41586-018-0590-4 PMC6642641

[20] Frankish A. , Diekhans M., Jungreis I., Lagarde J., Loveland J.E., Mudge J.M., Sisu C., Wright J.C., Armstrong J., Barnes I. et al. gencode 2021. Nucleic Acids Res. 2021; 49:D916–D923. 33270111 10.1093/nar/gkaa1087 PMC7778937

[21] O’Leary N.A. , Wright M.W., Brister J.R., Ciufo S., Haddad D., McVeigh R., Rajput B., Robbertse B., Smith-White B., Ako-Adjei D. et al. Reference sequence (RefSeq) database at NCBI: current status, taxonomic expansion, and functional annotation. Nucleic Acids Res. 2016; 44:D733–D745. 26553804 10.1093/nar/gkv1189 PMC4702849

[22] Morales J. , Pujar S., Loveland J.E., Astashyn A., Bennett R., Berry A., Cox E., Davidson C., Ermolaeva O., Farrell C.M. et al. A joint NCBI and EMBL-EBI transcript set for clinical genomics and research. Nature. 2022; 604:310–315. 35388217 10.1038/s41586-022-04558-8 PMC9007741

[23] Hsu F. , Kent W.J., Clawson H., Kuhn R.M., Diekhans M., Haussler D The UCSC known genes. Bioinformatics. 2006; 22:1036–1046. 16500937 10.1093/bioinformatics/btl048

[24] Cezard T. , Cunningham F., Hunt S.E., Koylass B., Kumar N., Saunders G., Shen A., Silva A.F., Tsukanov K., Venkataraman S. et al. The european variation archive: a FAIR resource of genomic variation for all species. Nucleic Acids Res. 2021; 50:D1216–D1220. 10.1093/nar/gkab960 PMC8728205 34718739

[25] Armstrong J. , Hickey G., Diekhans M., Fiddes I.T., Novak A.M., Deran A., Fang Q., Xie D., Feng S., Stiller J. et al. Progressive cactus is a multiple-genome aligner for the thousand-genome era. Nature. 2020; 587:246–251. 33177663 10.1038/s41586-020-2871-y PMC7673649

[26] Paten B. , Earl D., Nguyen N., Diekhans M., Zerbino D., Haussler D Cactus: algorithms for genome multiple sequence alignment. Genome Res. 2011; 21:1512–1528. 21665927 10.1101/gr.123356.111 PMC3166836

[27] Zoonomia Consortium A comparative genomics multitool for scientific discovery and conservation. Nature. 2020; 587:240–245. 33177664 10.1038/s41586-020-2876-6 PMC7759459

[28] The GTEx Consortium The GTEx consortium atlas of genetic regulatory effects across human tissues. Science. 2020; 369:1318–1330. 32913098 10.1126/science.aaz1776 PMC7737656

[29] Turakhia Y. , Thornlow B., Hinrichs A.S., De Maio N., Gozashti L., Lanfear R., Haussler D., Corbett-Detig R. Ultrafast sample placement on existing tRees (UShER) enables real-time phylogenetics for the SARS-CoV-2 pandemic. Nat. Genet. 2021; 53:809–816. 33972780 10.1038/s41588-021-00862-7 PMC9248294

[30] O’Toole Á. , Scher E., Underwood A., Jackson B., Hill V., McCrone J.T., Colquhoun R., Ruis C., Abu-Dahab K., Taylor B. et al. Assignment of epidemiological lineages in an emerging pandemic using the pangolin tool. Virus Evol. 2021; 7:veab064. 34527285 10.1093/ve/veab064 PMC8344591

[31] McBroome J. , Thornlow B., Hinrichs A.S., Kramer A., De Maio N., Goldman N., Haussler D., Corbett-Detig R., Turakhia Y. A daily-updated database and tools for comprehensive SARS-CoV-2 mutation-annotated trees. Mol. Biol. Evol. 2021; 38:5819–5824. 34469548 10.1093/molbev/msab264 PMC8662617

[32] Hammal F. , de Langen P., Bergon A., Lopez F., Ballester B. ReMap 2022: a database of human, mouse, drosophila and arabidopsis regulatory regions from an integrative analysis of DNA-binding sequencing experiments. Nucleic Acids Res. 2021; 50:D316–D325. 10.1093/nar/gkab996 PMC8728178 34751401

[33] Benson D.A. , Cavanaugh M., Clark K., Karsch-Mizrachi I., Lipman D.J., Ostell J., Sayers E.W. GenBank. Nucleic Acids Res. 2013; 41:D36–D42. 23193287 10.1093/nar/gks1195 PMC3531190

[34] Nurk S. , Koren S., Rhie A., Rautiainen M., Bzikadze A.V., Mikheenko A., Vollger M.R., Altemose N., Uralsky L., Gershman A. et al. The complete sequence of a human genome. Science. 2022; 376:44–53. 35357919 10.1126/science.abj6987 PMC9186530

[35] Nair S. , Barrett A., Li D., Raney B.J., Lee B.T., Kerpedjiev P., Ramalingam V., Pampari A., Lekschas F., Wang T. et al. The dynseq genome browser track enables visualization of context-specific, dynamic DNA sequence features at single nucleotide resolution genomics. 2022; bioRxiv doi:31 May 2022, preprint: not peer reviewed 10.1101/2022.05.26.493621. PMC10015500 36241719

[36] Yates A.D. , Achuthan P., Akanni W., Allen J., Allen J., Alvarez-Jarreta J., Amode M.R., Armean I.M., Azov A.G., Bennett R. et al. Ensembl 2020. Nucleic Acids Res. 2020; 48:D682–D688. 31691826 10.1093/nar/gkz966 PMC7145704

