# Supplementary material for: The UCSC Genome Browser database: 2023 update
Source: Nucleic Acids Res. 2022 Nov 24;51(D1):D1188–95. doi: 10.1093/nar/gkac1072 (PMC9825520; doi:10.1093/nar/gkac1072)
Supplement: gkac1072_Supplemental_File [file gkac1072_supplemental_file.docx]

| Track name | Assembly |
| --- | --- |
| Orphadata: Aggregated Data From Orphanet (1) | hg19, hg38 |
| Microarray Probesets | hg19, hg38 |
| dbSNP 155 (2) | hg19, hg38 |
| JARVIS: score to prioritize non-coding regions for disease relevance (3) | hg19, hg38 |
| HMC - Homologous Missense Constraint Score on PFAM domains | hg19, hg38 |
| gnomAD Genome Mutational Constraint (4) | hg38 |
| gnomAD Predicted Constraint Metrics (pLI and Z-scores) (5) | hg38 |
| The Gene Curation Coalition (GenCC) Annotations (6) | hg38 |
| MTR - Missense Tolerance Ratio Scores (7) | hg19 |
| MetaDome - Tolerance Landscape score  (8) | hg19 |

**Supplementary Table S1:** New and updated clinical tracks for hg19 and hg38.

| Track name | Assembly |
| --- | --- |
| GTEx High-Confidence cis-eQTLs from CAVIAR (9) | hg38 |
| GTEx V8 RNA-Seq Read Coverage by Tissue (10) | hg38 |
| GTEx cis-eQTL | hg38 |
| Cactus 241-way comparative genomics (11,12) | hg38 |
| Peripheral blood mononuclear cells (PBMC) from Hao et al 2020 (13) | hg38 |
| Colon single cell sequencing from Wang et al 2020 (14) | hg38 |
| Cerebral cortex single cell data from Velmeshev et al 2019 (15) | hg38 |
| Fetal Gene Atlas from Cao et al 2020 (16) | hg38 |
| Heart single cell RNA data from https://www.heartcellatlas.org (17) | hg38 |
| Ileum single cell sequencing from Wang et al 2020 | hg38 |
| Pancreas single cell sequencing from Baron et al 2016 (18) | hg38 |
| Muscle single cell data from De Micheli et al 2020 (19) | hg38 |
| Lung cells from from Travaglini et al 2020 (20) | hg38 |
| Liver single cell sequencing from MacParland et al 2018 (21) | hg38 |
| Kidney single cell data from Stewart et al 2019 (22) | hg38 |
| Placenta and decidua cells from from Vento-Tormo et al 2018 (23) | hg38 |
| Rectum single cell sequencing from Wang et al 2020 | hg38 |
| Skin single cell data from Sole-Boldo et al 2020 (24) | hg38 |
| Merged Cells: Single cell RNA expression levels cell types from many organs | hg38 |
| Tabula Sapiens single cell RNA data (25) | hg38 |
| ReMap Atlas of Regulatory Regions (26) | hg38, hg19, mm39, mm10, dm6 |
| JASPAR Transcription Factor Binding Sites (27) | hg38 hg19 mm39 mm10 |
| European Variation Archive (28) | mm39, mm10, danRer11, danRer10, +12 more |
| CRISPR Targets (29) | bosTau9 |

**Supplementary Table S2:** New and updated clinical tracks for hg19 and hg38.

| Track name | Assembly |
| --- | --- |
| GENCODE Genes v41 (knownGene) (30) | hg38 |
| GENCODE Genes v41 | hg19, hg38 |
| GENCODE Genes v40 | hg19, hg38 |
| GENCODE Genes v39 (knownGene) | hg38 |
| GENCODE Genes v39 | hg19, hg38 |
| GENCODE Genes v38 (knownGene) | hg38 |
| GENCODE Genes VM30 (knownGene) | mm39 |
| GENCODE Genes VM30 | mm39 |
| GENCODE Genes VM29 | mm39 |
| GENCODE Genes VM28 | mm39 |
| GENCODE Genes VM27 | mm39 |
| MANE Select v1.0 (31) | hg38 |
| NCBI RefSeq 109.20211119 (32) | hg38 |
| NCBI RefSeq 105.20220307 | hg19 |

**Supplementary Table S3:** New and updated gene model tracks on human and mouse. Tracks designated knownGene contain additional data associations and serve as the default genes track for the assembly.

| Track name |
| --- |
| Variants of Concern (VOC): Includes the latest WHO-designated VOC from <https://www.who.int/activities/tracking-SARS-CoV-2-variants> |
| Varskip Primers |
| Natural selection data from <https://observablehq.com/@spond/revised-sars-cov-2-analytics-page> |
| Omicron BA.4 and BA.5 |
| Galaxy ENA |
| Predicted Covid-drug resistance mutation positions (33) |
| ARTIC v4.1 (34) |
| ARTIC v4 |
| Omicron mutations |
| Problematic Sites (35) |
| SARS-CoV-2 Phylogeny (36) |
| UniProt Protein Annotations (37) |

**Supplementary Table S4:** List of new and updated tracks for the SARS-CoV-2 assembly.

| Track name | Assembly |
| --- | --- |
| DECIPHER CNVs (38) | hg38 |
| NHGRI Catalog of Published GWAS (39) | hg38, hg19, hg18 |
| GeneReviews (40) | hg38, hg19, hg18 |
| Locus Reference Genomic (LRG) / RefSeqGene Fixed Transcript Annotations (41) | hg38, hg19 |
| Orphadata: Aggregated Data From Orphanet | hg38, hg19 |
| ClinVar Variants (42) | hg38, hg19 |
| OMIM Genes & Phenotypes | hg38, hg19, hg18 |
| OMIM Allelic Variant Phenotypes | hg38, hg19, hg18 |
| Mastermind Variants (43) | hg38, hg19 |
| UniProt Protein Annotations | hg38, hg19 + 110 more |
| ClinGen Research (44) | hg38, hg19 |
| GRC Incident Database | hg38, hg19, mm10, mm9, danRer10, danRer7 |
| RefSeq genes from NCBI | hg38, hg19 |

**Supplementary Table S5:** List of tracks and corresponding assemblies belonging to our automatic pipelines that were updated since August 2021. These annotations update regularly without manual intervention.

| Hub name | Assembly |
| --- | --- |
| ReMap 2022 Regulatory Atlas | mm10, mm39, hg38, hg19, dm6, araTha1 |
| Golden Hamster genome (45) | MesAur2.0 |
| Bird Alignment (363 species): Zoonomia Project (46) | 363 bird assemblies |
| Mammal and Bird Alignment (605 species) (47) | 605 mammal and bird assemblies |
| EyeBrowse | hg19, bosTau6, canFam2, cavPor3, danRer6, danRer7, rn4, rheMac2, galGal3, hg17, hg18, mm9 |
| Singing mouse genome hub | steguina |
| Dfam database of TE families and annotations | hg38 |

**Supplementary Table S6:** New Genome Browser Public Hubs added in the last year.

**SUPPLEMENTARY REFERENCES**

1. Pavan,S., Rommel,K., Mateo Marquina,M.E., Höhn,S., Lanneau,V. and Rath,A. (2017) Clinical Practice Guidelines for Rare Diseases: The Orphanet Database. *PLoS One*, **12**, e0170365.

2. Sherry,S.T., Ward,M.-H., Kholodov,M., Baker,J., Phan,L., Smigielski,E.M. and Sirotkin,K. (2001) dbSNP: the NCBI database of genetic  variation. *Nucleic Acids Res*, **29**, 308–311.

3. Vitsios,D., Dhindsa,R.S., Middleton,L., Gussow,A.B. and Petrovski,S. (2021) Prioritizing non-coding regions based on human genomic constraint and sequence context with deep learning. *Nat Commun*, **12**, 1504.

4. Chen,S., Francioli,L.C., Goodrich,J.K., Collins,R.L., Wang,Q., Alföldi,J., Watts,N.A., Vittal,C., Gauthier,L.D., Poterba,T., *et al.* (2022) A genome-wide mutational constraint map quantified from variation in 76,156 human genomes. *bioRxiv*, [10.1101/2022.03.20.485034](https://doi.org/10.1101/2022.03.20.485034).

5. Exome Aggregation Consortium, Lek,M., Karczewski,K.J., Minikel,E.V., Samocha,K.E., Banks,E., Fennell,T., O’Donnell-Luria,A.H., Ware,J.S., Hill,A.J., *et al.* (2016) Analysis of protein-coding genetic variation in 60,706 humans. *Nature*, **536**, 285–291.

6. DiStefano,M.T., Goehringer,S., Babb,L., Alkuraya,F.S., Amberger,J., Amin,M., Austin-Tse,C., Balzotti,M., Berg,J.S., Birney,E., *et al.* (2022) The Gene Curation Coalition: A global effort to harmonize gene-disease evidence resources. *Genet Med*, **24**, 1732–1742.

7. Silk,M., Petrovski,S. and Ascher,D.B. (2019) MTR-Viewer: identifying regions within genes under purifying selection. *Nucleic Acids Res*, **47**, W121–W126.

8. Wiel,L., Baakman,C., Gilissen,D., Veltman,J.A., Vriend,G. and Gilissen,C. (2019) MetaDome: Pathogenicity analysis of genetic variants through aggregation of homologous human protein domains. *Hum Mutat*, **40**, 1030–1038.

9. The Genotype-Tissue Expression (GTEx) project (2013) *Nat Genet*, **45**, 580–585.

10. Melé,M., Ferreira,P.G., Reverter,F., DeLuca,D.S., Monlong,J., Sammeth,M., Young,T.R., Goldmann,J.M., Pervouchine,D.D., Sullivan,T.J., *et al.* (2015) The human transcriptome across tissues and individuals. *Science*, **348**, 660–665.

11. Paten,B., Earl,D., Nguyen,N., Diekhans,M., Zerbino,D. and Haussler,D. (2011) Cactus: Algorithms for genome multiple sequence alignment. *Genome Res*, **21**, 1512–1528.

12. Zoonomia Consortium (2020) A comparative genomics multitool for scientific discovery and conservation. *Nature*, **587**, 240–245.

13. Hao,Y., Hao,S., Andersen-Nissen,E., Mauck,W.M., Zheng,S., Butler,A., Lee,M.J., Wilk,A.J., Darby,C., Zager,M., *et al.* (2021) Integrated analysis of multimodal single-cell data. *Cell*, **184**, 3573-3587.e29.

14. Wang,Y., Song,W., Wang,J., Wang,T., Xiong,X., Qi,Z., Fu,W., Yang,X. and Chen,Y.-G. (2019) Single-cell transcriptome analysis reveals differential nutrient absorption functions in human intestine. *J Exp Med*, **217**, e20191130.

15. Velmeshev,D., Schirmer,L., Jung,D., Haeussler,M., Perez,Y., Mayer,S., Bhaduri,A., Goyal,N., Rowitch,D.H. and Kriegstein,A.R. (2019) Single-cell genomics identifies cell type–specific molecular changes in autism. *Science*, **364**, 685–689.

16. Cao,J., O’Day,D.R., Pliner,H.A., Kingsley,P.D., Deng,M., Daza,R.M., Zager,M.A., Aldinger,K.A., Blecher,R., Zhang,F., *et al.* (2020) A human cell atlas of fetal gene expression. *Science*, **370**, eaba7721.

17. Litviňuková,M., Talavera-López,C., Maatz,H., Reichart,D., Worth,C.L., Lindberg,E.L., Kanda,M., Polanski,K., Heinig,M., Lee,M., *et al.* (2020) Cells of the adult human heart. *Nature*, **588**, 466–472.

18. Baron,M., Veres,A., Wolock,S.L., Faust,A.L., Gaujoux,R., Vetere,A., Ryu,J.H., Wagner,B.K., Shen-Orr,S.S., Klein,A.M., *et al.* (2016) A Single-Cell Transcriptomic Map of the Human and Mouse Pancreas Reveals Inter- and Intra-cell Population Structure. *Cell Syst*, **3**, 346-360.e4.

19. De Micheli,A.J., Spector,J.A., Elemento,O. and Cosgrove,B.D. (2020) A reference single-cell transcriptomic atlas of human skeletal muscle tissue reveals bifurcated muscle stem cell populations. *Skelet Muscle*, **10**, 19.

20. Travaglini,K.J., Nabhan,A.N., Penland,L., Sinha,R., Gillich,A., Sit,R.V., Chang,S., Conley,S.D., Mori,Y., Seita,J., *et al.* (2020) A molecular cell atlas of the human lung from single cell RNA sequencing. *Nature*, **587**, 619–625.

21. MacParland,S.A., Liu,J.C., Ma,X.-Z., Innes,B.T., Bartczak,A.M., Gage,B.K., Manuel,J., Khuu,N., Echeverri,J., Linares,I., *et al.* (2018) Single cell RNA sequencing of human liver reveals distinct intrahepatic macrophage populations. *Nat Commun*, **9**, 4383.

22. Stewart,B.J., Ferdinand,J.R., Young,M.D., Mitchell,T.J., Loudon,K.W., Riding,A.M., Richoz,N., Frazer,G.L., Staniforth,J.U., Braga,F.A.V., *et al.* (2019) Spatio-temporal immune zonation of the human kidney. *Science*, **365**, 1461–1466.

23. Vento-Tormo,R., Efremova,M., Botting,R.A., Turco,M.Y., Vento-Tormo,M., Meyer,K.B., Park,J.-E., Stephenson,E., Polański,K., Goncalves,A., *et al.* (2018) Single-cell reconstruction of the early maternal–fetal interface in humans. *Nature*, **563**, 347–353.

24. Solé-Boldo,L., Raddatz,G., Schütz,S., Mallm,J.-P., Rippe,K., Lonsdorf,A.S., Rodríguez-Paredes,M. and Lyko,F. (2020) Single-cell transcriptomes of the human skin reveal age-related loss of fibroblast priming. *Commun Biol*, **3**, 188.

25. Schaum,N., Karkanias,J., Neff,N.F., May,A.P., Quake,S.R., Wyss-Coray,T., Darmanis,S., Batson,J., Botvinnik,O., Chen,M.B., *et al.* (2018) Single-cell transcriptomics of 20 mouse organs creates a Tabula Muris. *Nature*, **562**, 367–372.

26. Hammal,F., de Langen,P., Bergon,A., Lopez,F. and Ballester,B. (2021) ReMap 2022: a database of Human, Mouse, Drosophila and Arabidopsis regulatory regions from an integrative analysis of DNA-binding sequencing experiments. *Nucleic Acids Res*, **50**, D316–D325.

27. Castro-Mondragon,J.A., Riudavets-Puig,R., Rauluseviciute,I., Berhanu Lemma,R., Turchi,L., Blanc-Mathieu,R., Lucas,J., Boddie,P., Khan,A., Manosalva Pérez,N., *et al.* (2021) JASPAR 2022: the 9th release of the open-access database of transcription factor binding profiles. *Nucleic Acids Res*, **50**, D165–D173.

28. Cezard,T., Cunningham,F., Hunt,S.E., Koylass,B., Kumar,N., Saunders,G., Shen,A., Silva,A.F., Tsukanov,K., Venkataraman,S., *et al.* (2021) The European Variation Archive: a FAIR resource of genomic variation for all species. *Nucleic Acids Res*, **50**, D1216–D1220.

29. Haeussler,M., Schönig,K., Eckert,H., Eschstruth,A., Mianné,J., Renaud,J.-B., Schneider-Maunoury,S., Shkumatava,A., Teboul,L., Kent,J., *et al.* (2016) Evaluation of off-target and on-target scoring algorithms and integration into the guide RNA selection tool CRISPOR. *Genome Biol*, **17**, 148.

30. Harrow,J., Frankish,A., Gonzalez,J.M., Tapanari,E., Diekhans,M., Kokocinski,F., Aken,B.L., Barrell,D., Zadissa,A., Searle,S., *et al.* (2012) GENCODE: The reference human genome annotation for The ENCODE Project. *Genome Res.*, **22**, 1760–1774.

31. Morales,J., Pujar,S., Loveland,J.E., Astashyn,A., Bennett,R., Berry,A., Cox,E., Davidson,C., Ermolaeva,O., Farrell,C.M., *et al.* (2022) A joint NCBI and EMBL-EBI transcript set for clinical genomics and research. *Nature*, **604**, 310–315.

32. Pruitt,K.D. (2004) NCBI Reference Sequence (RefSeq): a curated non-redundant sequence database of genomes, transcripts and proteins. *Nucleic Acids Research*, **33**, D501–D504.

33. Sedova,M., Jaroszewski,L., Iyer,M. and Godzik,A. (2022) Monitoring for SARS-CoV-2 drug resistance mutations in broad viral populations Bioinformatics.

34. artic-ncov2019/primer_schemes/nCoV-2019/V4 at master · artic-network/artic-ncov2019 (2022) *GitHub*.

35. Turakhia,Y., Thornlow,B., Gozashti,L., Hinrichs,A.S., Haussler,D. and Corbett-Detig,R. Stability of SARS-CoV-2 Phylogenies.

36. roblanf and Mansfield,R. (2020) roblanf/sarscov2phylo: 13-11-20. [10.5281/zenodo.4289383](https://doi.org/10.5281/zenodo.4289383).

37. Reorganizing the protein space at the Universal Protein Resource (UniProt) (2012) *Nucleic Acids Res*, **40**, D71–D75.

38. Firth,H.V., Richards,S.M., Bevan,A.P., Clayton,S., Corpas,M., Rajan,D., Vooren,S.V., Moreau,Y., Pettett,R.M. and Carter,N.P. (2009) DECIPHER: Database of Chromosomal Imbalance and Phenotype in Humans Using Ensembl Resources. *Am J Hum Genet*, **84**, 524–533.

39. Potential etiologic and functional implications of genome-wide association loci for human diseases and traits (2022) [10.1073/pnas.0903103106](https://doi.org/10.1073/pnas.0903103106).

40. Adam,M.P., Everman,D.B., Mirzaa,G.M., Pagon,R.A., Wallace,S.E., Bean,L.J., Gripp,K.W. and Amemiya,A. eds. (1993) GeneReviews® University of Washington, Seattle, Seattle (WA).

41. Dalgleish,R., Flicek,P., Cunningham,F., Astashyn,A., Tully,R.E., Proctor,G., Chen,Y., McLaren,W.M., Larsson,P., Vaughan,B.W., *et al.* (2010) Locus Reference Genomic sequences: an improved basis for describing human DNA variants. *Genome Med*, **2**, 24.

42. Landrum,M.J., Lee,J.M., Benson,M., Brown,G., Chao,C., Chitipiralla,S., Gu,B., Hart,J., Hoffman,D., Hoover,J., *et al.* (2016) ClinVar: public archive of interpretations of clinically relevant variants. *Nucleic Acids Res*, **44**, D862–D868.

43. Chunn,L.M., Nefcy,D.C., Scouten,R.W., Tarpey,R.P., Chauhan,G., Lim,M.S., Elenitoba-Johnson,K.S.J., Schwartz,S.A. and Kiel,M.J. (2020) Mastermind: A Comprehensive Genomic Association Search Engine for Empirical Evidence Curation and Genetic Variant Interpretation. *Front Genet*, **11**, 577152.

44. Rehm,H.L., Berg,J.S., Brooks,L.D., Bustamante,C.D., Evans,J.P., Landrum,M.J., Ledbetter,D.H., Maglott,D.R., Martin,C.L., Nussbaum,R.L., *et al.* (2015) ClinGen — The Clinical Genome Resource. *N Engl J Med*, **372**, 2235–2242.

45. Ishino,K., Hasuwa,H., Yoshimura,J., Iwasaki,Y.W., Nishihara,H., Seki,N.M., Hirano,T., Tsuchiya,M., Ishizaki,H., Masuda,H., *et al.* (2021) Hamster PIWI proteins bind to piRNAs with stage-specific size variations during oocyte maturation. *Nucleic Acids Research*, **49**, 2700–2720.

46. Feng,S., Stiller,J., Deng,Y., Armstrong,J., Fang,Q., Reeve,A.H., Xie,D., Chen,G., Guo,C., Faircloth,B.C., *et al.* (2020) Dense sampling of bird diversity increases power of comparative genomics. *Nature*, **587**, 252–257.

47. Armstrong,J., Hickey,G., Diekhans,M., Fiddes,I.T., Novak,A.M., Deran,A., Fang,Q., Xie,D., Feng,S., Stiller,J., *et al.* (2020) Progressive Cactus is a multiple-genome aligner for the thousand-genome era. *Nature*, **587**, 246–251.
